# Evidence of a Novel Mechanism for Partial γ-Secretase Inhibition Induced Paradoxical Increase in Secreted Amyloid β Protein

**DOI:** 10.1371/journal.pone.0091531

**Published:** 2014-03-21

**Authors:** Eliza Barnwell, Vasudevaraju Padmaraju, Robert Baranello, Javier Pacheco-Quinto, Craig Crosson, Zsolt Ablonczy, Elizabeth Eckman, Christopher B. Eckman, Viswanathan Ramakrishnan, Nigel H. Greig, Miguel A. Pappolla, Kumar Sambamurti

**Affiliations:** 1 Department of Neurosciences, Medical University of South Carolina, Charleston, South Carolina, United States of America; 2 Biomedical Research Institute of New Jersey, MidAtlantic Neonatology Associates and Atlantic Health System, Morristown, New Jersey, United States of America; 3 Department of Ophthalmology, Medical University of South Carolina, Charleston, South Carolina, United States of America; 4 Department of Biostatistics, Medical University of South Carolina, Charleston, South Carolina, United States of America; 5 Drug Design & Development Section, Translational Gerontology Branch, Intramural Research Program, National Institute on Aging, Baltimore, Maryland, United States of America; 6 Department of Neurology, University of Texas Medical Branch, Galveston, Texas, United States of America; IIBB/CSIC/IDIBAPS, Spain

## Abstract

BACE1 (β-secretase) and α-secretase cleave the Alzheimer's amyloid β protein (Aβ) precursor (APP) to C-terminal fragments of 99 aa (CTFβ) and 83 aa (CTFα), respectively, which are further cleaved by γ-secretase to eventually secrete Aβ and Aα (a.k.a. P3) that terminate predominantly at residues 40 and 42. A number of γ-secretase inhibitors (GSIs), such as N-[N-(3,5-Difluorophenacetyl-L-alanyl)]-S-phenylglycine *t*-butyl ester (DAPT), have been developed with the goal of reducing Aβ to treat Alzheimer's disease (AD). Although most studies show that DAPT inhibits Aβ in a dose-dependent manner several studies have also detected a biphasic effect with an unexpected increase at low doses of DAPT in cell cultures, animal models and clinical trials. In this article, we confirm the increase in Aβ40 and Aβ42 in SH-SY5Y human neuroblastoma cells treated with low doses of DAPT and identify one of the mechanisms for this paradox. We studied the pathway by first demonstrating that stimulation of Aβ, a product of γ-secretase, was accompanied by a parallel increase of its substrate CTFβ, thereby demonstrating that the inhibitor was not anomalously stimulating enzyme activity at low levels. Secondly, we have demonstrated that inhibition of an Aβ degrading activity, endothelin converting enzyme (ECE), yielded more Aβ, but abolished the DAPT-induced stimulation. Finally, we have demonstrated that Aα, which is generated in the secretory pathway before endocytosis, is not subject to the DAPT-mediated stimulation. We therefore conclude that impairment of γ-secretase can paradoxically increase Aβ by transiently skirting Aβ degradation in the endosome. This study adds to the growing body of literature suggesting that preserving γ-secretase activity, rather than inhibiting it, is important for prevention of neurodegeneration.

## Introduction

For those afflicted, AD destroys cognitive function over time during the later stages of life. According to recent records, it currently afflicts 5.4 million Americans at a cost of $200 billion this year in healthcare alone (www. Alz.org). Estimates of AD suggest that more than one quarter of the elderly population older than 90 years-of age experience this devastating neurodegenerative disease and about 5% suffer from early onset forms of the disease [1]. AD is characterized by brain deposits of extracellular senile plaques (SP) containing Aβ42 and intracellular neurofibrillary tangles (NFTs) containing the microtubule associated protein, tau (MAPT) [2]. Studies have identified early onset familial AD (FAD) mutations in APP and in presenilin 1 (PS1) and 2 (PS2) that display the entire spectrum of AD pathology establishing the role of the Aβ pathway in the disease process [3], [4]. The convergence of FAD mutations on APP, accumulation of SP and *in vitro* neurotoxicity of Aβ is the foundation of the widely recognized ‘amyloid hypothesis’ of AD [5], [6]. This hypothesis has fueled extensive work on its biogenesis and turnover.

**Figure 1:**
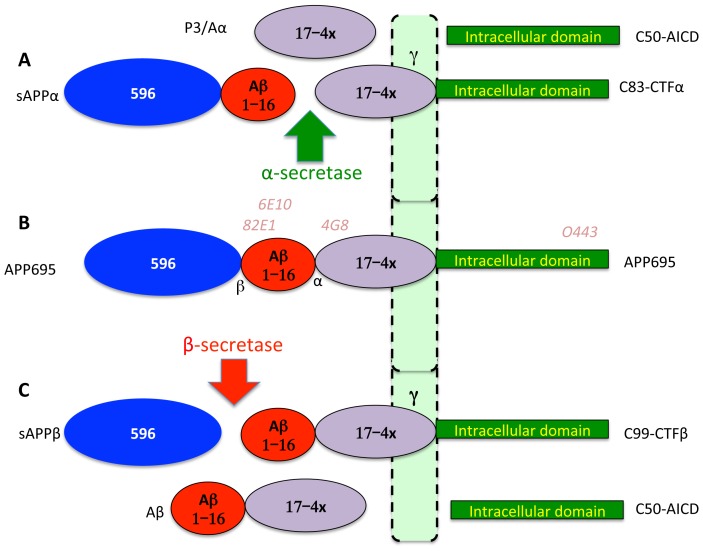
Key APP processing pathways. APP is a type-1 integral-membrane glycoprotein with a large ectodomain a single transmembrane domain and a short intracellular domain (B). While it exists in multiple forms, we are using neuroblastoma cells overexpressing the neuronal 695 aa form. In this form, the ectodomain includes a region of 596 aa that is cleaved and secreted by BACE1 called sAPPβ (Blue ellipse) leaving behind the CTFβ of 99 aa (C). The Aβ sequence starts with the first 16 aa, which is released with the 596 aa after cleavage by α-secretase to sAPPα of 612 aa, and CTFα of 83 aa (A). The presenilin-containing multisubunit γ-secretase cleaves CTFα (A) and CTFβ (C) to secreted proteins of 3 kDa (Aα) and 4 kDa (Aβ), respectively. Although multiple intramembrane intermediate forms of Aβ and P3 are reported, the major secreted forms terminate at residue 40 followed at much lower levels by residue 42 of the Aβ sequence. Numerous studies have demonstrated that most FAD mutations preferentially increase Aβ42. Antibodies used in the study are indicated above the APP schematic (B). Domains of APP are not drawn to scale but colors are consistent in all figures. Note that we are using Aα instead of the more common P3 or Aβ 17–40/42 to maintain processing pathway consistency that makes it easier for the non-expert.

Major findings in AD research are that APP is a large protein of 695–770 amino acids (aa) generated by alternative splicing of a single gene on chromosome 21 [7]. All APP forms are type-I integral membrane glycoproteins with a large N-terminal ectodomain, a single transmembrane domain and a short cytoplasmic tail (Fig 1). BACE1, a type-I membrane bound aspartyl protease, processes APP to generate CTFβ starting at the Aβ sequence, which is further processed by γ-secretase comprising a complex of four proteins containing PS1/PS2, APH1, PEN2 and Nicastrin to generate largely Aβ40 and secondly (5–10%) Aβ42 and an intracellular fragment, AICD of 50 aa [8]–[11]. However, most APP is cleaved at an alternate site between residues 16 and 17 of Aβ by α-secretase to generate a large secreted protein sAPPα and intracellular CTFα, which is then processed by γ-secretase to Aα. Most FAD mutations on PS1, PS2 and APP appear to selectively increase the levels of Aβ42 without affecting Aβ40, but there are exceptions (*e.g.*APP670NL mutation increases Aβ40 and Aβ42 [5]). However, screening through the mutations in Alzgene reveals that different mutations perform differently with some showing increases and others reductions in individual Aβ forms [12] (http://www.alzforum.org/mutations). Nevertheless, all the FAD mutations deposit Aβ as a defining criterion for AD showing that they somehow foster amyloid accumulation.

The importance of Aβ reduction to potentially mitigate AD is further reinforced by the recent identification of an APP variant that reduces AD risk and lowers Aβ [13]. Given the widely accepted central role for Aβ as a trigger for AD, the major focus of industry has been to eliminate SP by immunotherapy or to inhibit Aβ generation via treatment with BACE1 inhibitors or GSIs. However, the recent failure of a major trial of the GSI, Semagacestat, leading to premature trial termination highlights our poor understanding of its role in AD pathogenesis [2], [14]. Here we show that—contrary to expectation—a prototypic GSI, DAPT, actually elevates, rather than lowers, both Aβ40 and Aβ42 secreted into cell culture media. This increase in secreted Aβ is distinct from the accumulation of longer membrane-bound Aβ intermediates such as the ζ form that increase intracellularly upon DAPT but not L-685,458 treatment [11]. Although this type of increase in Aβ has been previously reported for multiple GSIs by several groups [15]–[19], this finding is not universal (e.g. [20]) and numerous studies have only detected a differential rise in Aβ42 levels [3], [21], [22]. This phenomenon has been considered a rebound effect and is absent for some of the potent GSIs [23]. Importantly, changes in APP CTFs, the substrates for γ-secretase, were not evaluated under conditions that stimulate Aβ production, making it unclear whether this is an effect of anomalous increase of γ-secretase activity or just a property of some of the compounds in use. In this study, we have investigated the dose- response relationship of Aβ40 and Aβ42 production in response to DAPT treatment in an undifferentiated human neuroblastoma cell line, SH-SY5Y, transfected with wild type human APP695 and have examined mechanisms of Aβ increase. Our results indicate that DAPT treatment leads to a bypass of at least one Aβ degrading enzyme, ECE, to increase the yield of the secreted Aβ peptide. Consistent with this finding, we do not observe the stimulation of Aβ in Chinese Hamster Ovary (CHO) cells that lack ECE. Since many studies of APP processing use CHO for their analysis, the lack of ECE may explain the widespread reports of dose-dependent inhibition of Aβ with most GSIs.

## Materials and Methods

### Reagents and antibodies

SH-SY5Y cells transfected with APP695 (SH-SY5Y-APP) and its culture conditions were as described previously [24]. CHO cells similarly transfected with APP695 were a gift from Dr. Todd Golde. All culture media and antibiotics were from Thermo-Fisher Scientific (Waltham, MA) or Invitrogen Corporation (Carlsbad, CA). Fetal bovine serum (FBS) was from Atlanta Biologics Corporation (Flowery Branch, GA). DAPT, O443 antibody against the C-terminal 20 residues of APP within its intracellular domain, WO2 against Aβ1-16 and the Western and chemiluminescent substrate for horseradish peroxidase (HRP) were from Calbiochem-Millipore Corporation (Billerica, MA). 82E1, an Aβ N-terminal end-specific antibody, and the Aβ1-40 sandwich ELISA kit were from IBL-America (Minneapolis, MN). ELISA assays for Aβ1-42 (Innogenetics, Ghent, Belgium), sAPP (R&D systems; Minneapolis, MN) or in house equivalents of all the ELISA assays were carried out as described previously [25], [26]. 4G8-Biotin against Aβ 17–24 (Covance; Princeton, NJ) was used to detect all Aβ forms. Phosphoramidon, an inhibitor of Thermolysin, Neprilysin (NEP) and Endothelin Converting Enzyme (ECE) was from peptide international. Precast Criterion™ XT 4-10% Bis-Tris gels and MES running buffer (BioRad Corporation) and nitrocellulose membranes (Whatman, Piscataway, NJ) were used for Western blot analysis. The signals were captured using an Alpha Innotech FluorChem™ system and quantified using the associated AlphaEase software.

### Cell culture

SH-SY5Y-APP cells were cultured in Dulbecco's Modified Eagle's Medium/Nutrient Mixture F12 (DMEM-F12) (Thermo scientific) medium with 10% heat inactivated FBS and 1% penicillin-streptomycin as described previously [24]. Cells were plated at 3×10^5^ cells per well in 6 well dishes and incubated for 24 h in DMEM-F12-10% FBS medium. Cultures were then treated with a range DAPT or vehicle (DMSO) in triplicates for each experiment. In some studies, parallel sets of cultures were treated with 100 μM PA and a range of DAPT. Media collected at indicated times were aliquoted and assayed for changes in APP metabolism as described below. Cells were lysed in ice-cold lysis buffer (50 mM Tris-HCL, pH 8.0, 150 mM NaCl, 1%NP40) and processed for Western blot analysis or protein assays. Pilot studies showed that inclusion of protease inhibitor cocktails did not change the profile of APP and its metabolites by either ELISA or Western blot analysis. These inhibitors were therefore not included for the study.

### ELISA assays for Aβ and Aα

Aβ40 and Aβ42 levels were measured by sensitive and specific sandwich ELISA assays that use end-specific antibodies to capture the Aβ40 and Aβ42 C-terminal cut ends without binding full-length APP or alternative Aβ fragments. Detection antibodies used in the assay were end-specific antibodies against the Aβ N-terminal 5 residues 3D6 (Innogenetics, biotin labeled) or 82E1-HRP (IBL-America) for the 42 and 40 kits, respectively to ensure that only full-length Aβ starting at position 1 is measured. Similarly plates coated with Aβ40 and Aβ42 specific capture antibodies were used for detection with 4G8-Biotin to detect all forms of Aβ and Aα generated after γ-secretase processing such as Aβ1-40/42, Aβ 11-40/42 and 17-40/42 with the last form defined as Aα generated from CTFα. In our discussion, we are ignoring products other than Aβ1-40/42 and 17-40/42 as minor and treat the value obtained from subtraction of the 4G8 ELISAs and the 1-40/42 ELISAs as Aα. Cell culture medium samples were applied to the plates and incubated for 2 h at room temperature, treated with the detection antibodies for 1 h at room temperature and washed. In the case of the biotinylated 3D6 and 4G8 antibodies, the plates were treated with HRP-labeled Streptavidin provided with the kits for 30 min at RT while this was not needed for 82E1-HRP. The plates were then washed with the provided wash buffer, developed with the HRP substrate (chromogen), terminated with 0.9N sulfuric acid and absorbance values read spectrophotometrically at 450 λ using a M5 multimodal plate reader (Molecular Devices).

### Western blotting

CTFα, CTFβ, and full-length APP levels in cell lysates and sAPPα in media were determined by quantitative Western blotting. After the experimental treatment, the cells were washed with PBS and lysed with lysis buffer (1%NP40, 50 mM Tris-HCL, pH 8.0, 150 mM NaCl) and subjected to SDS-polyacrylamide gel electrophoresis using 12% Bis-Tris precast gels in MES buffer, blotted onto nitrocellulose membranes, blocked with 10% NCS for 1 h at RT, washed with TBST (6×10 min) and probed with the O443 against the final 20 intracellular residues of APP or 82E1 antibodies against the cleaved N-terminus of CTFβ/Aβ described earlier. For the quantification of sAPPα the cell culture media were processed in a similar manner and probed with WO2, an antibody against Aβ residues 1–16. The blots were developed using the chemiluminescent substrate for HRP and the signals were captured using a Fluorochem HD detector (Alpha Innotech) and quantified using the included AlphaEase software.

### Statistical analysis

Statistical analysis of quantitative data was carried out using PROC GLM in SAS. Post-hoc comparisons were made using the Tukey-Kramer method. For some comparisons a simple t-test was utilized. Please note that the Microsoft Excel software was used to automatically draw the trendlines in scatter plots in relevant figures for graphic representation, but all statistics were calculated from the raw data and were not influenced by the curves.

## Results

### DAPT-stimulates both Aβ40 and Aβ42 in SH-SY5Y-APP cells

**Figure 2:**
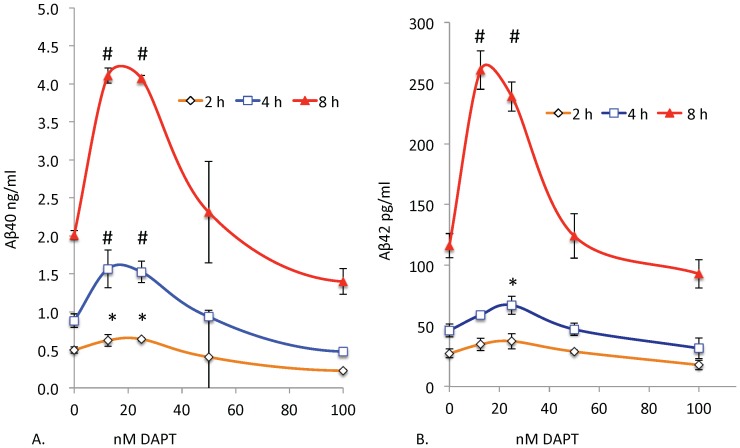
Low doses of DAPT unexpectedly increase Aβ40 and Aβ42 levels. SH-SY5Y-APP cells were cultured in six-well dishes and treated with either vehicle (DMSO) or DAPT at 12.5 to 1000 nM for 2 (orange), 4 (blue) and 8 h (red). Media were analyzed by sensitive and specific sandwich ELISA assays that use end-specific antibodies to specifically capture the Aβ40 (A) and Aβ42 (B) and were detected using the end-specific antibodies for the Aβ N-terminus (3D6, Innogenetics). After Tukey-Kramer adjustment the P values for left panel (A) showed highly significant stimulation of Aβ40 at 12.5 and 25 nM (p<0.0001) at 8 h and significant stimulation at 2 and 4 h. A similar stimulation was also observed for Aβ42 at 12.5 and 25 nM (p<0.0002). Inhibition at a dose of 1 μM was highly significant (p<0.0001) for Aβ40 and 42 as expected (Data not shown). For all graphs significance indicated by symbols # p<0.01 and * p<0.05.

We treated SH-SY5Y-APP cells with a range of DAPT concentrations for 8 h and determined their effects on Aβ levels in the culture media. Although DAPT is a potent GSI, at low doses below 100 nM, it unexpectedly stimulates both Aβ40 and Aβ42 (Fig 2A, B). However, at higher doses (1000 nM), DAPT strongly inhibits both Aβ40 and Aβ42 (not shown) as consistently reported in numerous publications [20]. Stimulation is observed at 2, 4 and 8 h with the same relative dose-response with stimulation peaks at 12.5 and 25 nM in the continuous presence of the drug (Fig 2). If this were due to a rebound effect caused by accumulation of substrate with inhibitor, followed by degradation of the drug during the incubation period, one would expect the stimulation concentration to shift with time as the drug degrades. However, the drug continues to stimulate and inhibit with similar doses over time, suggesting that the effect is mediated by continuous presence of the active inhibitor. We also observed the same pattern of stimulation followed by inhibition with other known potent GSIs such as compound E and to a lesser degree with L411,575 and L-685,458 (data not shown).

### Low doses of DAPT does not stimulate γ-secretase activity

**Figure 3:**
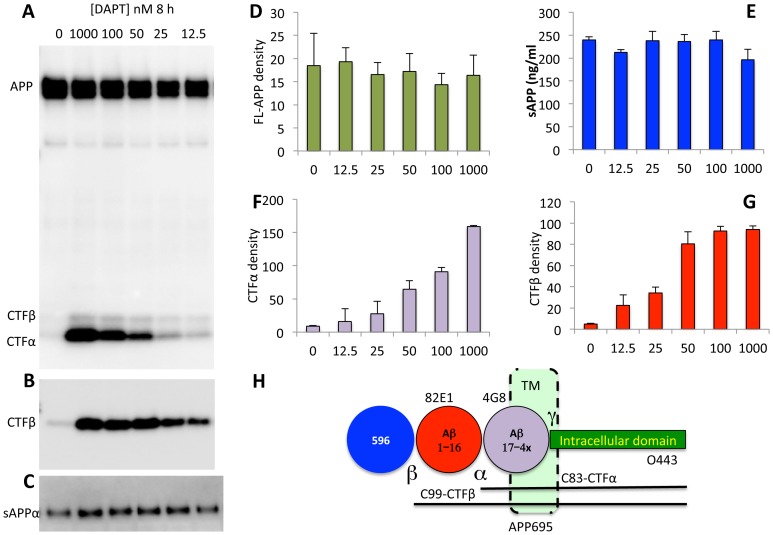
DAPT induced increase in Aβ does not alter APP processing. Western blots of cell lysates of SH-SY5Y cells from Fig 2 were detected in triplicates using the O443 antibody (A) and 82E1 (B); and media were analyzed with the WO2 antibody against Aβ1-16 (C) as described in Materials and Methods. Full-length APP from A (D), CTFα from A (F) and CTFβ from B (G) were quantified using the AlphaEase software and total sAPP (E; measured by an ELISA assay from R&D systems) were plotted as bar graphs. Full-length and secreted APP in panels D and E are not different from vehicle-treated controls. Tukey-Kramer analysis shows that there is significant dose-dependent increase in CTFβ (p<0.0001) and CTFα (p<0.0001) with DAPT treatment. Note that CTFβ reaches saturation at 50 nM DAPT whereas O443 does not saturate even at 100 nM. Panel H: Schematic of the APP695 protein as in Fig 1.

To determine whether DAPT stimulation of secreted Aβ levels is due to activation of γ-secretase by the GSI, we treated cells with a range of DAPT doses and determined relative APP, CTFα and CTFβ levels. Both CTFα (Fig 3A, F) and CTFβ (Fig 3B, G) increase in a dose-dependent manner even at these low doses, without significant effects on full-length APP (Fig 3A, D) or sAPPα (Fig 3C, E), demonstrating that the substrate of γ-secretase – CTFβ and CTFα– increase even at DAPT doses that actually increase rather than reduce Aβ with treatment (Fig 3). Based on these findings, we concluded that the DAPT-mediated stimulation of Aβ is not due to anomalous enzyme activation, but due to changes in Aβ after its biogenesis. It is technically possible that DAPT also inhibits an Aβ degrading enzyme, in addition to its production, but we did not observe Aβ protection by degradation assays in vitro (Baranello et al, Manuscript in preparation). Failure of DAPT to inhibit Aβ degradation in cell lysates and media was also reported previously [19].

A secondary observation is that quantitative comparison of CTFα (Fig 3F) and CTFβ (Fig 3G) suggests that the former continues to increase at all doses, whereas CTFβ saturates at 50 nM DAPT. Since BACE1 is known to cleave APP after endocytosis [27], [28], its kinetics may indicate that CTFβ turnover in the endosome may be more rapidly inhibited, presumably due to predicted quicker uptake of the inhibitor into endosomes and slower distribution to the trans-Golgi network and secretory vesicles that are constantly being replaced. Unfortunately, there is no reliable and simple method to test this possibility due to the transient nature of this interaction.

### PA overcomes the DAPT-induced stimulation of Aβ production

It was previously reported that PA, a potent inhibitor of neutral endopeptidase, NEP, and ECE strongly increases Aβ production from several cell lines, primary brain cultures and in live animals [29], [30]. One study also used more selective ECE inhibitors to demonstrate that the activity was mediated by ECE rather than other related enzymes and that it was resistant to thiorphan, which inhibits NEP without also inhibiting ECE [24], [31]. The studies also demonstrated that an endosome form of ECE was responsible for degrading Aβ at an acid pH in a manner coupled with production, and that it failed to degrade externally added Aβ [24]. ECE has a property of only degrading small peptides and does not even degrade Aβ42 as well as Aβ40 and is therefore not expected to cleave CTFα and CTFβ [29]. Based on this literature and the localization of BACE1 cleavage, we hypothesized that Aβ will be degraded in the recycling endosome by ECE and that CTFβ and possibly other intermediates such as longer forms of Aβ will transiently accumulate in the endosome in the presence of DAPT to be recycled intact to the cell surface either directly or via the trans-Golgi network (TGN) as in the hypothetical model (see discussion). Since the TGN and cell surface will contain more enzyme under conditions where γ-secretase is only partially inhibited and since there will be plenty of time for the recycled precursors to be processed, we expect it to be converted to more Aβ. However, this new Aβ should be in the secretory compartment safe from ECE degradation.

**Figure 4:**
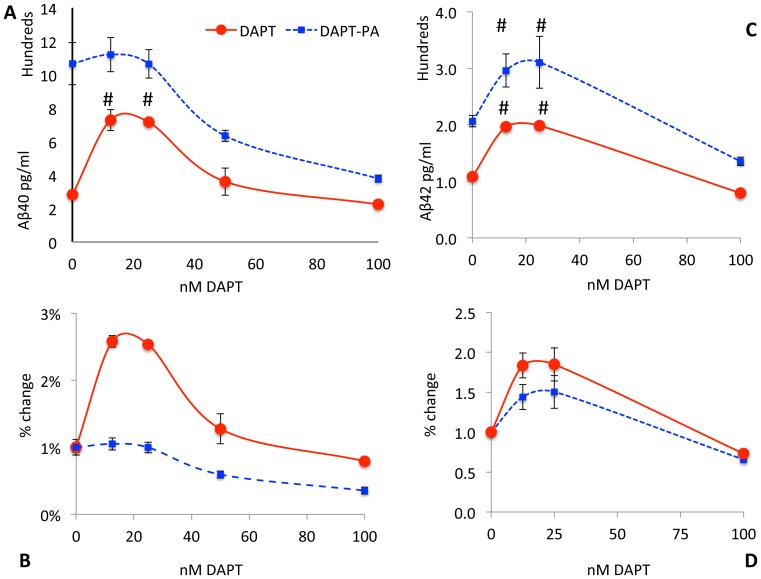
PA treatment mitigates Aβ stimulation. SH-SY5Y cells were treated with a combination of PA and DAPT (Blue squares and dashed lines) or DAPT alone (Red circles, continuous line) and Aβ40 (A) or Aβ42 (C) were plotted as raw data in pg/ml or as percent change (C, D). The change from the 0 DAPT control show that stimulation by low level GSI treatment is completely blocked by PA treatment for Aβ40 (B) but remained at 1.4 fold for Aβ42 (D). Nevertheless, the DAPT-induced stimulation of Aβ42 at 12.5 and 25 nM was also significantly (p = 0.03) attenuated (C, D). The DAPT-mediated stimulation in PA-treated cells is 4-fold for Aβ40 compared to two fold for Aβ42, suggesting that ECE contributes more substantially to Aβ40 turnover than Aβ42, but DAPT increases Aβ42 equally by avoiding other unidentified degrading enzymes by the same mechanism.

**Figure 5:**
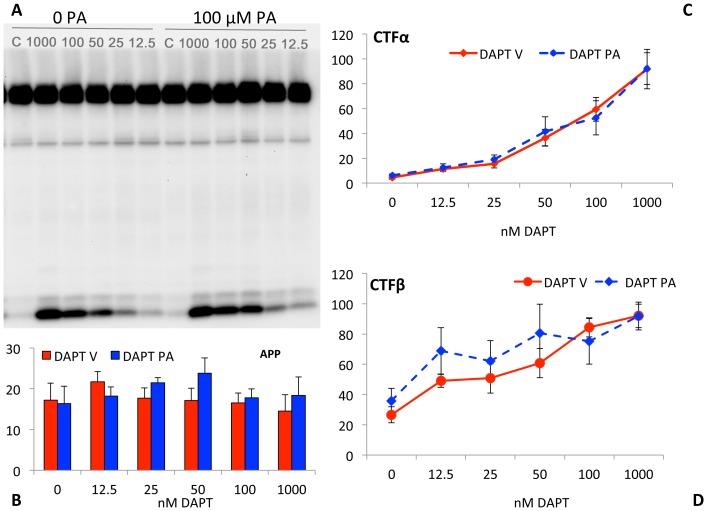
PA treatment does not affect APP processing. SH-SY5Y-APP695 cells were treated with 0 to 1,000 nM DAPT in the absence and presence of 100 μM PA and cell lysates were analyzed as in Fig 3. Panel A is a representative Western blot with O443 showing APP (top band), CTFβ (Faint lower band) and CTFα (dark lowest band). The quantified band intensities for APP (B), CTFα (C) and CTFβ (D) show the changes observed with DAPT treatment in the presence (blue bar and dotted lines with solid diamonds) or absence of PA (red bars, solid red circles). Note that the labels on the x-axis are not drawn to scale to include the 1000 nM DAPT data. Tukey-Kramer analysis show that there is significant dose-dependent increase in CTFβ (p<0.002) and CTFα (p<0.0001) with DAPT treatment, but there is no significant change in either CTFβ (p>0.2, 0.5) or CTFα (p>0.8, 0.9).

In the absence of DAPT, PA treatment substantially increased the levels of both Aβ40 (277%; Fig 4A and Aβ42) (90%; Fig 4C) yield. This PA treatment, either alone or in combination with DAPT, did not change levels of either CTFα (Fig 5A, C) or CTFβ (Fig 5A, D). Note that although CTFβ appears to increase slightly (35% without DAPT and 40% with 12.5 nM DAPT) in Fig 5D, these changes are nonsignificant (p>0.3) and unable to explain the 4-fold increase in Aβ40 and 2 fold increase in Aβ42 with DAPT treatment given that the increase in the ratio of CTFβ from 0 to 12.5 nM DAPT in PA vs. vehicle treatment is only (5%). Thiorphan, a potent NEP inhibitor, does not affect Aβ levels or DAPT-mediated stimulation in SH-SY5Y-APP (Baranello and Sambamurti, unpublished observations), suggesting that the Aβ degradation we are observing is driven by ECE.

In the presence of PA, DAPT showed a dose-dependent inhibition of Aβ40 starting at a higher level, while in the absence of PA, the curve started at a much lower level without DAPT and then rose for the two lower DAPT doses (Fig 4A). In the presence of PA, only slight stimulation (5%) was observed at the lowest DAPT dose of 12.5 nM, and this change was not significant (p>0.9) while this change was 158% (p<0.0001) in the absence of PA (Fig 4B). Parallel analysis of Aβ42 showed a significant, but smaller increase with PA treatment (p = 0.003), suggesting that unlike Aβ40, the ECE-degraded pool of Aβ42 is smaller. Nevertheless, Aβ42 is protected to a similar extent by low doses of DAPT, suggesting that other unidentified Aβ42-specific degrading enzymes are similarly bypassed by the treatment. This finding is consistent with previous reports that Aβ40 is a better substrate for ECE than Aβ42 [29], [30]. DAPT treatment in the presence of PA increases Aβ42 by 40% (p = 0.09), compared to a 75% increase (P = 0.02) without PA. In conclusion, PA inhibits degradation of both Aβ40 and Aβ42 and DAPT stimulation of both forms of Aβ is strongly attenuated by PA. The effect is much greater for Aβ40, which is also better substrate for ECE.

**Figure 6:**
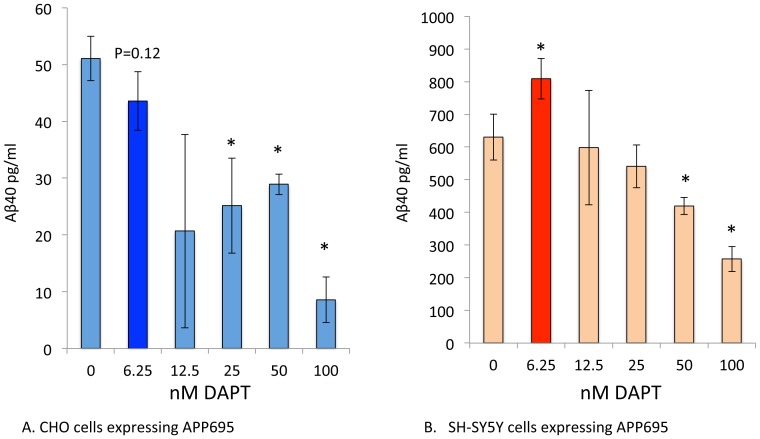
CHO cells fail to display the DAPT induced stimulation of Aβ. CHO cells and SH-SY5Y cells expressing human APP695 were treated with 6.25, 12.5, 25, 50 and 100 nM DAPT for 2 h. DAPT induced the consistent increase in Aβ40 at 6.25 nM (<0.05), but the CHO-APP695 failed to show any stimulation of Aβ40 but showed a trend towards reduction (P = 0.12) instead. Aβ42 was not detectable in these transfected CHO- APP695 cells.

Knocking out ECE using siRNA was attempted by Pacheco-Quinto and Eckman (unpublished observations), but detected nonspecific changes even with control siRNAs. However, to genetically confirm the findings, we tested whether Aβ will be reduced in a cell line known to lack ECE expression and therefore not sensitive to PA mediated increase in Aβ. One such cell line is CHO as discussed earlier in introduction. In transfected CHO, we did not have sufficient expression to detect Aβ42, but were able to adequately measure Aβ40, which shows more robust PA-dependent effects in SH-SY5Y-APP. Side-by-side studies show that DAPT stimulates Aβ40 in SH-SY5Y cells (Fig 6B) but fails to do so in CHO cells, which only secretes lower levels of Aβ40 in a dose-dependent manner (Fig 6A). These data provide additional confirmation that the effects are mediated by ECE rather than an unknown secondary effect of PA. However, one may still argue that CHO is not SH-SY5Y lacking ECE, but a different cell line from a different organism. While these studies cannot solve the issue of perfect comparison, previous studies have demonstrated the phenomena in humans, guinea pigs and primary mouse brain cultures, suggesting that the phenomenon is not species limited and at least using a hamster cell should not prevent the phenomenon [15]-[19]. The bypass phenomenon therefore must involve ECE, at least for Aβ40 and possibly other Aβ degrading enzymes that are absent in CHO cells.

The results therefore support the hypothesis that DAPT may not directly inhibit Aβ degradation and tricks the cell to shift production to compartments that lack ECE. However, more elaborate follow up studies are required to determine whether CTFβ or other intermediates are involved in the protected pool and to identify the exact subcellular compartments involved.

### Aα, the product of α-and γ-secretase cleavage, is not stimulated by DAPT treatment

Unlike BACE1, a number of studies suggest that α-secretase activity resides in the secretory pathway and on the cell surface [28], [32]–[34]. We therefore predicted that the α-secretase derived CTFα would not need to go through the endosomal compartment to be processed by γ-secretase to Aα. Thus, our prediction was that we would not obtain a DAPT-induced increase in Aα although we should continue to obtain an increase in Aβ.

**Figure 7:**
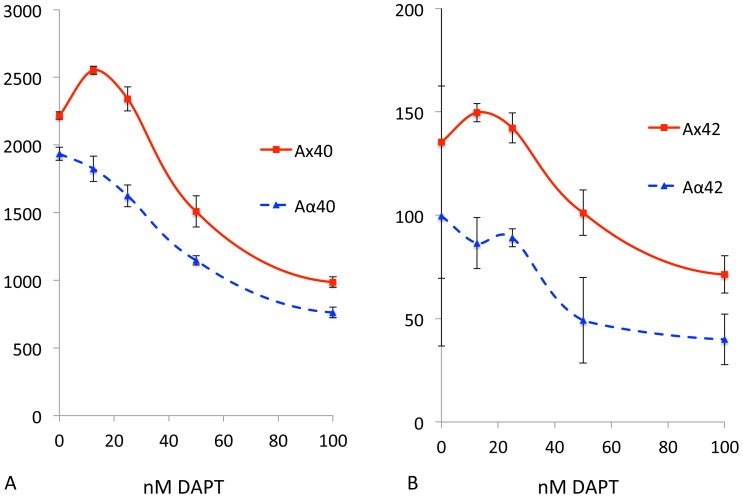
The secreted APP fragment generated by α and γ secretase is not stimulated by DAPT. SH-SY5Y cells were treated with a combination of DAPT and media were analyzed using a combination of ELISA assays capturing secreted fragments ending at Aβ residue 40 and 42 and detecting with 4G8 to determine the total Ax40 and Ax42 (red circles, solid line) and the Aβ40 and Aβ42 values were subtracted to obtain Aα40 and Aα42 (blue triangles, dotted lines). Although similar trends were observed for Ax42 and Ax42 Tukey-Kramer analysis gave highly significant P values for increase in Ax40 (<0.05; A) but not for Ax42 (>0.7). (<0.0002; B). In contrast Aα40 showed a dose-dependent inhibition (p<0.0001) for all doses except 12.5 nM (p = 0.054) with no stimulation at 12.5 and 25 nM DAPT. Aα42 showed similar trends, but the values were not significant. Furthermore, elimination of an outlier control revealed a small, but significant increase in Aα42 (not shown).

In order to measure Aα, we used a strategy of capturing all the secreted peptides terminating at residues 40 and 42 and on plates coated with end-specific antibodies for the cleaved protein and detecting with the biotinylated 4G8 antibody against residues 17–24 of Aβ to identify all species longer than Aα17–40 (Ax40) and 17–42 (Ax42). We also measured Aβ40/Aβ42 levels and subtracted the values from Ax40/Ax42 to obtain Aα40 and Aα42, ignoring the minor forms such as Aβ11-40/42 that would also be included. Aα40 levels were five-fold higher than Aβ40 and Aα42 were two-fold higher than Aβ42 in the absence of DAPT. These data are consistent with the approximately five fold higher levels of CTFα vs CTFβ presented as substrates to γ-secretase in SH-SY5Y (data not shown). DAPT treatment reduces Aα40 (Fig 7A dotted line) in a dose-dependent manner while Ax40, which includes Aβ and Aα, showed the expected stimulation in the products. Aα42 also showed a similar trend without any stimulation, but the dose-dependent reduction is not statistically significant. If we were to eliminate an outlier among the control values, the data even showed some stimulation that cannot be adequately explained at this time. In summary, the data show that both species of Aα are reduced in a dose-dependent manner and once again the phenomenon is more robust and easily detected for the shorter Aα40 than for Aα42.

## Discussion

Amyloid deposition is a defining feature of AD. Numerous investigators have demonstrated that oligomeric forms of Aβ are neurotoxic and therefore conclude that accumulation of Aβ is the major trigger in AD pathogenesis [35]–[39]. Thus, there has been a major effort to develop agents that reduce Aβ production for the prevention and treatment of AD. This effort has been further justified by the finding that numerous mutations on APP, PS1 and PS2 increase Aβ42 production even in cell cultures [37].

Understanding the failure of Aβ homeostasis that leads to the slow accumulation of amyloid as plaques and cerebrovascular amyloid is therefore critical to the understanding of AD pathogenesis and to prevent toxicity from developing before it becomes too late to treat by amyloid lowering strategies. Important steps in this pathway include changes in expression of APP [40], [41], BACE1 [42]–[44], or any of the γ-secretase subunits [45]. Importantly, complex regulation of γ-secretase provides multiple potential bottlenecks that may impair its activity ranging from the expression of various subunits, their splicing events, allelic variations, use of alternative subunits such as PS1 and PS2 or APH1a or APH1b [46] as well as protein trafficking pathways to the cell surface and endosomes [33], [47]–[53]. Similarly, alternatively spliced forms of Nicastrin appear to increase AD risk only in the presence of the ApoE-ε4 allele [54], [55]. A number of previous studies have shown that alternatively spliced forms of PS1 lacking exon 8 also lose a critical transmembrane domain aspartate residue (D257) that is believed to be responsible for catalytic activity [56], [57]. However transfection studies using this naturally occurring alternatively spliced form did not reduce Aβ production although it impaired the processing of Notch, a key developmental regulator of neurogenesis [58], [59]. Further, these studies showed that APP-CTFs do accumulate under these conditions indicating impairment of γ- secretase processing of APP that did not yet get revealed as reduction in Aβ levels was nevertheless present. Further studies in SH-SY5Y and similar cell types are needed to understand these natural phenomena in the light of the novel findings in this article, as these γ-secretase impairments may be captured as stimulation of Aβ instead.

From the start, there has been a mechanistic debate on whether the PS1/2 mutations provide a gain of function or loss of function to explain this increase in Aβ42 levels [19], [57], [60]–[65]. The first available drugs developed to reduced Aβ were GSIs that targeted the final step in Aβ generation and there is extensive literature ranging from in vitro assays and cell cultures to clinical trials [66]. Unfortunately, several GSIs have recently failed in AD clinical trials (www.alzforum.org) with most discussions focused on their off target detrimental, primarily on Notch signaling [67], [68]. The effects of GSIs and FAD mutations are however far from fully characterized despite numerous studies on their properties. In this study, we have demonstrated that a widely used and well-characterized GSI, DAPT, increases Aβ40 and Aβ42 at low concentration, albeit it inhibits them at high levels. This paradoxical finding does not extend to the CTF substrates of γ-secretase, which increase with inhibitor dose as predicted. In addition, CTFα processing to Aα40 is not subject to the same stimulation as Aβ40. Finally, we find that PA increases Aβ40 and attenuates the DAPT mediated increase in Aβ levels.

**Figure 8:**
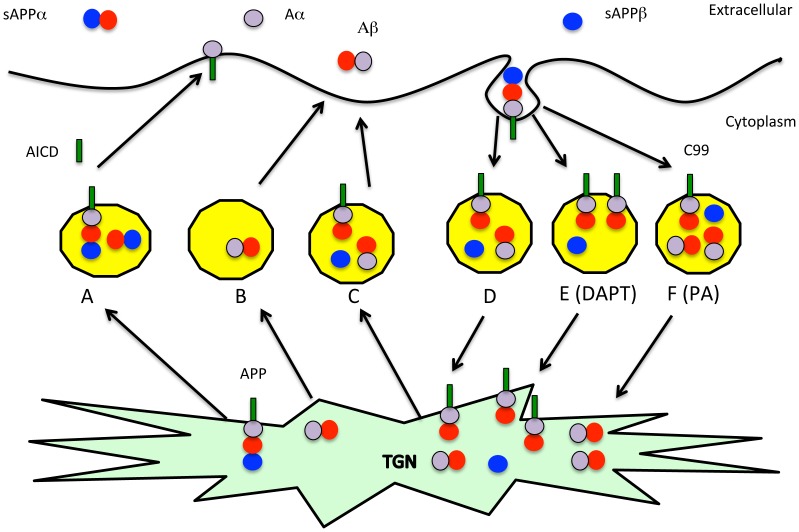
Model showing the mechanism for increase in Aβ with γ-secretase impairment. The hypothetical model derives from the known cellular localization of Aβ production and turnover pathways to explain the unexpected and contrasting changes in APP metabolites with DAPT treatment [32], [78]–[80]. APP shown with a black circle to the β-secretase site, an open circle to represent Aβ1-16 to the junction of the α-secretase cleavage site and a grey circle for the remainder of Aβ starting at position 17. Secreted sAPPα is shown as a joined black and open circle, Aβ as an open and grey circle, Aα as a grey circle and CTFα/β as a combination of circles with a tail embedded in the membrane. APP is predominantly processed by α-secretase in the secretory pathway from the trans-Golgi network (TGN) to the cell surface generating CTFα, which is processed by γ-secretase in the secretory pathway (A). On the other hand, BACE1 cleaves APP in the endocytosis pathway to C99 where γ-secretase generates some Aβ (D). The Aβ (B) and remaining unprocessed C99 (C) are transported to the surface where the residual C99 is converted to Aβ by γ-secretase and both pools of Aβ are secreted into the medium. Aβ, but not C99 is degraded primarily by ECE in the endosome (D, E). Inhibition by GSI increases the C99 pool in the endosome (E) but this C99 is further processed during the recycling step where it reaches the cell surface either directly or via the TGN to generate Aβ that now escapes degradation in the endosome. One can also increase the secreted Aβ by inhibiting ECE with PA, but this increase does not affect CTFβ (F).

It has been previously established that BACE1 processing of APP occurs in the endocytic pathway whereas α-secretase cleaves in the secretory pathway [27], [28], [32], [69]–[72]. Furthermore, ECE mediated degradation has been tentatively mapped to the recycling endosome where BACE1 and γ-secretase cleave APP to generate Aβ, although a caveat remains that ECE levels in untransfected cells are too small to be detected by Western blot analysis [24], [73], [74]. Consistent with this cellular localization, we propose that BACE1 cleaves APP primarily in the endosome where γ-secretase is also present to generate Aβ40 and Aβ42 and ECE degrades the nascent Aβ before it is released to reduce its yield in the medium (Fig 8D). In the presence of low levels of DAPT, CTFβ and longer Aβ intermediate forms of 45–49 residues accumulate inside the vesicle and remain associated with membranes (Fig 8E). Since ECE is known to require small peptide substrates, these membrane bound intermediates in Aβ biogenesis should not get degraded [30] allowing them to survive the recycling to the trans-Golgi network and cell surface where the bulk of cellular γ-secretase activity is present and can therefore process them to secreted Aβ40 and Aβ42 forms. In the presence of PA, Aβ degradation is impaired, so it does not make a difference whether CTFβ or Aβ accumulate in the recycling vesicle. These studies therefore provide strong evidence for increase in Aβ upon γ-secretase impairment. Supporting evidence for such an involvement is seen in an APP-expressing mouse model that also includes a PS1 FAD mutation where we see that deposition of Aβ is accompanied by accumulation of CTFα and CTFβ [75]. In conclusion, these studies are consistent with our previously proposed hypothesis that impairment of γ-secretase may be a key mechanism for AD pathogenesis and may include the failure of membrane protein turnover that then leads to neuronal dysfunction [2], [4], [10]. Consistent with this theory, γ-secretase impairment can lead to blood-retinal barrier dysfunction as demonstrated in failure of tight junctions in endothelial cell and retinal pigmented epithelial cell cultures [76], [77]. The finding that impairment of γ-secretase also elevates Aβ suggests that increased Aβ and SPs may be markers of γ-secretase impairment. However, rather than inhibiting γ-secretase, one may need to identify and avoid physiological and environmental agents that impair its activity to avoid AD. Furthermore, potential AD treatments may need to focus on preserving or stimulating rather than inhibiting γ-secretase function.
